# Comparison of affected sibling-pair linkage methods to identify gene × gene interaction in GAW15 simulated data

**DOI:** 10.1186/1753-6561-1-s1-s66

**Published:** 2007-12-18

**Authors:** Emma K Larkin, Nathan J Morris, Yali Li, Nora L Nock, Catherine M Stein

**Affiliations:** 1 Department of Epidemiology and Biostatistics, Case Western Reserve University, Triangle Building, Suite 260, 11400 Euclid Avenue, Cleveland, Ohio 44106 USA

## Abstract

Non-parametric linkage methods have had limited success in detecting gene by gene interactions. Using affected sibling-pair (ASP) data from all replicates of the simulated data from Problem 3, we assessed the statistical power of three approaches to identify the gene × gene interaction between two loci on different chromosomes. The first method conditioned on linkage at the primary disease susceptibility locus (DR), to find linkage to a simulated effect modifier at Locus A with a mean allele sharing test. The second approach used a regression-based mean test to identify either the presence of interaction between the two loci or linkage to the A locus in the presence of linkage to DR. The third method applied a conditional logistic model designed to test for the presence of interacting loci. The first approach had decreased power over an unconditional linkage analysis, supporting the idea that gene × gene interaction cannot be detected with ASP data. The regression-based mean test and the conditional logistic model had the lowest power to detect gene × gene interaction, possibly because of the complex recoding of the tri-allelic DR locus for use as a covariate. We conclude that the ASP approaches tested have low power to successfully identify the interaction between the DR and A loci despite the large sample size, which may be due to the low prevalence of the high-risk DR genotypes. Additionally, the lack of data on discordant sibships may have decreased the power to identify gene × gene interactions.

## Background

Linkage analysis methods to identify gene × gene interactions in complex diseases have been developed [1-4], however, in the absence of already characterized candidate genes, their ability to identify epistasis is unknown. Moreover, analyses from the Genetic Analysis Workshop (GAW) 14 simulated data further support the difficulty in locating gene × gene interaction [5]. The GAW15 simulated rheumatoid arthritis (RA) data set affords another opportunity to compare the statistical power of three non-parametric linkage approaches using affected sibling pairs (ASPs) to identify gene × gene interactions between two unlinked loci: 1) locus DR, which was simulated to represent the risk of DRB1 locus of HLA on RA, and 2) locus A, which was simulated as an effect modifier on DR.

First, we examined a mean test variant of the conditional methodology presented by Cox et al. [1]. The motivation behind this methodology is that correlations between identity-by-descent (IBD) allele sharing at unlinked loci can be used to identify the relationship between loci. We adapted this methodology to the mean test for linkage at another locus by excluding ASPs with no evidence for linkage to the first locus.

We also examined the power and type I error of two other covariate based approaches to detect epistasis with varying covariate coding schemes of the genotyped locus (DR). The first is a regression-based mean test that can be used to test for the presence of gene × environment interactions in ASPs [2]. By treating the DR locus as an "environmental variable," it is possible to use this method to identify gene × gene interaction. Second, we explored the conditional logistic model developed by Olson and colleagues [4,6], which is an alternative parameterization of the LOD score model presented by Risch [7]. Significant increases in linkage between a baseline model without covariates and a model with the DR locus as a covariate suggest epistasis between the DR locus and the locus where linkage is assessed.

## Methods

### Sample

The simulated data set from GAW15 Problem 3 consists of a 5-cM microsatellite genome scan for each of 100 replicates, in which each replicate represents a random sample of 1500 ASPs with RA and their parents (four-person pedigrees). Data from all replicates were analyzed with researchers unblinded to the simulation parameters. The DR locus on chromosome 6 was simulated as the primary disease susceptibility locus with additional genetic and environmental factors affecting the risk of disease. Only Locus A on chromosome 16 was simulated as an effect modifier on the risk of RA due to the DR locus. Thus, it was used as the test locus for gene × gene interaction. The DR locus has three alleles: X, 1, and 4, with prevalences 0.65, 0.1, and 0.25, respectively. The A locus is diallelic and acts in a dominant fashion with a prevalence of 0.3 for risk allele "A". Assuming Hardy-Weinberg proportions at the DR locus and holding other risk factors constant, the marginal risk of RA due to the DR locus in individuals with the A allele at locus A is 5.2, which decreases to 3.5 in individuals who are homozygous for the low-risk "a" allele at Locus A.

Multipoint allele sharing from ASPs was determined using GENIBD (S.A.G.E. v5.2). Parental genotype data were recoded to missing for deceased individuals. Various coding schemes for the covariate DR locus were examined including: 1) the X allele under an additive genetic model; 2) the 4 allele under an additive model; and, 3) a linear combination of the covariates based entirely on the simulated risk levels provided in the solutions. The "linear" coding for each individual given their DR locus genotype was constructed as follows: 1) "X/X" genotype was assigned a value of 0; 2) "X/1" or "X/4" genotypes were assigned a value of 1; 3) "4/4" genotype was assigned a value of 2 and, 4) "1/4" or "1/1" genotypes were assigned a value of 3. This coding scheme was designed to capture in a simple fashion the increased risk associated with the DR1 and DR4 alleles, on the basis of the values of the risk multipliers which are 0.8, 1, 2, and 6, respectively.

### Statistical analysis

The percent of replicates in which the *p*-value for linkage on chromosome 16 was less than 0.05 was used to estimate power. Type I error was determined by taking the chromosomes with no simulated disease or quantitative trait loci and averaging the number of times a replicate exceeded the threshold value of the test statistic at the *α *= 0.05 level. Within each replicate, the locus with the highest proportion of alleles shared IBD within a 20-cM region of the DR locus was selected as representing the point with most significant evidence for linkage to the DR locus because linkage can be detected as far as 20 cM away from the causal locus [8].

#### 1) Conditional Method

Let *π *be the mean proportion of alleles shared IBD between ASPs at a marker locus. The mean test compares the average amount of allele sharing IBD at a marker locus to the expected value of *π *= 0.5. Any excess of allele sharing across all sibling pairs is believed to be due to a disease susceptibility locus. A traditional *t*-statistic can be computed to compare the observed allele sharing to the null value of 0.5 with *n *- 1 degrees of freedom. A genome scan using the mean test was repeated, selecting only ASPs in which the proportion of alleles shared IBD was greater than or equal to a cut-off value, thus extracting families with evidence for linkage to the DR locus. Three cut-off values were selected: 0.5, 0.7, and 0.9. By testing various subsets, we were effectively applying 0,1 weights proposed by Cox et al. [1] to select ASPs with evidence for allele sharing at the DR locus. ASPs contributing to linkage at the DR locus should also be linked to the A locus if interactions exists [1]. Analyses were performed using the mean test in SIBPAL (S.A.G.E. v. 5.2).

#### 2) Mean Interaction Test Method

Alternatively, an intercept only (*π*_0_) regression model is equivalent to the mean test, where *ε*_*i *_represent the errors for each ASP *i *that are normally distributed with mean 0 and variance *σ*^2^: *π*_*i *_= *π*_0 _+ *ε*_*i *_[2]. A test for linkage only can be conducted by a likelihood ratio test or by Wald test ((*π*_0 _- 0.5)/(s.e.(*π*_0_)))^2^. The regression-based mean test is extended to allow for the inclusion of a mean centered covariate *X*_*i *_that captures the joint values of the sibling pairs at the DR locus as described above [2]. In this analysis we used the mean-corrected average of the sibling values:

$$\pi_{i}=\pi_{0}+\beta (X_{i}-\bar{X})+\varepsilon_{i}.$$

A likelihood ratio test was conducted with *π*_0 _= 0.5 and *β *= 0 against the alternative that *π*_0 _> 0.5 or *β *≠ 0 with a resultant test statistic that is distributed as a 50:50 mixture of *χ*^2^_1 _and *χ*^2^_2 _[2]. In addition, we performed a Wald test of *β *= 0 against the alternative that *β *≠ 0 using SAS v. 8.1, which can be interpreted as a test for interaction.

#### 3) Conditional Logistic Model Method

LODPAL (S.A.G.E. v 5.2) implements the conditional logistic model [4], which estimates *λ*_*i*_, the recurrence risk ratio for an affected sibling pair that shares *i *alleles IBD (for *i *= 0, 1, or 2) with the constraint that *λ*_2 _= 3.634*λ*_1 _- 2.634 [6]. The effect of covariates was assessed by estimating *λ*_1 _= exp(*β *+ *γx*), where *β *measures the genetic effect at the marker and *x *is the sib-pair covariate. The DR locus was included as a covariate by summing each sibling pair's individual values using the aforementioned genotype codes that were mean-corrected. A likelihood ratio test was conducted by comparing 2ln10 times the difference in LOD scores between models with and without the covariate to a $\chi_{1}^{2}$ distribution. For this distribution to be valid, loci with LOD = 0 were removed from the analysis and the denominators for calculations of type I error and power were adjusted accordingly. This adjustment is due to the fact that LODPAL rounds any negative LOD score up to 0.

## Results

**Figure 1:**
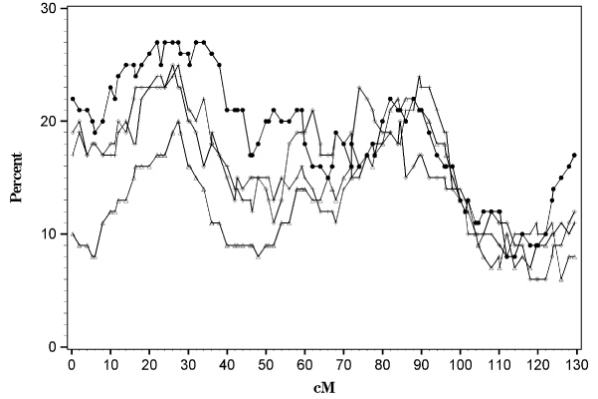
**Mean test for linkage on chromosome 16 using various subsets of ASPs**. The percent of significant results (at *α *= 0.05) is plotted against the centimorgan (cM) location. Solid circles represent the entire data set, while the plus signs, diamonds, and triangles represent those ASPs in which *π *at the DR locus was greater than 0.5, 0.7, and 0.9, respectively.

The conditional method using the subsetting approach did not provide additional power to detect linkage above and beyond using the entire data set to detect a main linkage effect of Locus A. Figure 1 shows that selecting a subset of ASPs with evidence for linkage to the DR locus did not vary by the arbitrary cut-points chosen. At 26 cM (the approximate location of the A locus) only 27 of the replicates detected linkage at an *α *= 0.05 level using all ASPs in each replicate. Restricting the sample sizes in each replicate by selecting ASPs with the proportion of alleles shared IBD at the DR locus greater than or equal to 0.5 resulted in slightly decreased power to detect linkage at the A locus than the complete data set (24%). DR allele sharing cutoffs of 0.7 or 0.9 did not increase power to detect linkage (24% and 20%, respectively). The type I error of these tests averaged, 10%, 9%, 9%, and 13% for cutpoints of 0.5, 0.7, 0.9, and the complete data set, respectively. To examine heterogeneity, we restricted the sample to those sib pairs with allele sharing less than 0.3 and 0.1, and the power again barely exceeded the type I error rate (data not shown).

**Figure 2:**
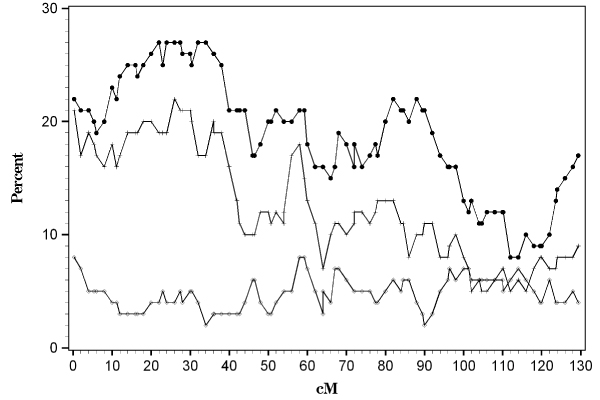
**Mean interaction test on chromosome 16**. The percent of significant results (at *α *= 0.05) is plotted against the centimorgan (cM) location. Solid circles represent the model with no covariate; plus signs indicate the joint test of *π *and *β*; and the diamonds represent the model with the interaction term (*β*) only.

Similarly, the mean interaction test had limited power to detect evidence for linkage to the A locus or evidence for a significant interaction using a test of the *β *coefficient. Figure 2 shows the percent of replicates that were detected across chromosome 16 for the ASP covariate sum that models the DR4 allele additively. The power of the test for linkage was 18% at the A locus and the power of the interaction term by itself was low (4%). Type I error was 5% and 9% for the interaction term alone and the joint test of linkage, respectively. The other models which used an alternative coding scheme for the covariate coding the DR locus (as described in the Methods section) produced similar results.

The conditional logistic methodology did not detect the interaction at the A locus, with the power ranging from 1 to 9% in the 40-cM region around the A locus. This power never exceeded the type I error. Even coding the covariates in a manner that mimicked the actual simulated risk parameters through a linear coding scheme did not improve the power to detect linkage.

## Discussion

None of the methods we examined had enough power at a type I error rate of *α *= 0.05 to detect linkage to the A locus in the presence of linkage to DR. There was no difference between the conditional method, which used a restricted sample, and the mean test and conditional logistic models, demonstrating that this lack of power was not due to insufficient sample size. Our results were quite similar to those of Brock et al. [9], who analyzed the GAW14 data. Of the 36 multipoint models that used the conditional logistic model, the highest power achieved was 34%. These results support the theoretical work by Vieland and Huang, which suggests that the establishment of epistasis in ASP data is impossible due to insufficient penetrance structure [10]. Using Gauderman and Siegmund's mean test [2], we found that the power of the test of the linkage-only model (*π *> 0.5) was greatest, followed by the joint test of *π *and *β*, and that the interaction only model had no less than 10% power. The power to detect interaction only is generally much lower than the power to detect linkage when allowing for interaction [3], so our results are not surprising. Furthermore, the work of Elston et al. [11] suggests that discordant pairs are necessary to detect gene × gene interaction, which was further demonstrated using simulated data [12]. Ultimately, our analyses are limited by the fact that these statistical definitions may not reflect biological reality in real data.

Our analyses of the GAW15 data also illustrate the challenges of testing for gene × gene interaction for complex diseases. First, we found that our analysis was hampered by the complexity of the simulated model. While the interaction was simulated to be large for certain genotypes, the prevalence of those genotypes was low. For example, the largest simulated multiplicative interaction between DR and the "A" allele of locus A was the DR4/DR4 genotype; however, the prevalence of DR4/DR4 was approximately 0.1. Gauderman and Siegmund demonstrate that, assuming a prevalence of exposure is at least 0.5, the increased risk must be greater than 3 to have sufficient power to detect gene × "environment" interaction [2]. In our analyses, we used the DR locus as the "environmental exposure"; thus, the low prevalence of the high risk genotype combinations likely affected our inability to detect the gene × gene interaction. The power of the conditional logistic model (in LODPAL) is greatest when using a dichotomous risk categorization as the covariate [3]; thus, we suspect that the reason we did not observe an increase in power when we used the DR locus as the covariate was because of the low frequency of the high risk DR genotypes. Second, a challenge in this data was that the DR locus was tri-allelic. Holmans [3] also discusses situations in which there are varying levels of risk associated with the candidate locus, or when certain genotypes may confer their increased risk in conjunction with different genotypes at the test locus. In these cases, the coding of the covariate is not trivial, and incorrect recoding may greatly reduce power. These complexities are certainly true of the simulated relationship between DR and A, and likely explain our loss of power. Finally, these results also raise another issue regarding the importance of the definition of the main effect and how main effects should be incorporated into such an analysis [13]. The first methodology that conditions on allele sharing at the first locus detects interaction that is a departure from the multiplicative penetrance model. An implicit assumption in detecting this kind of interaction is the presence of joint Hardy-Weinberg proportions or gametic phase equilibrium. While the A and the DR loci are on different chromosomes, it is possible that additional allelic association not due to linkage disequilibrium exists. Schaid et al. also indicate that for the mean interaction test to be valid the two loci must be uncorrelated in the general population [14], which would be true if joint Hardy-Weinberg equilibrium holds. This limitation holds for the conditional method, as well.

## Conclusion

In summary, we observed that the most commonly used current methods for detecting gene × gene interaction in ASP data had low power to detect the interaction between DR and A in the simulated data set. This lack of power is likely due to the lack of information in ASP data compared to having discordant pair data and/or the low prevalence of the high-risk DR genotypes and the complex nature of the simulated risk multipliers. Although complicated, this simulated data probably more accurately depicts real complex disease data; thus, we believe further research on linkage methods that can more powerfully detect epistasis while minimizing type I error is warranted.

## Competing interests

The author(s) declare that they have no competing interests.
